# Clustering and Interpretability of Residential Electricity Demand Profiles

**DOI:** 10.3390/s25072026

**Published:** 2025-03-24

**Authors:** Sarra Kallel, Manar Amayri, Nizar Bouguila

**Affiliations:** Concordia Institute for Information Systems Engineering, Concordia University, Montreal, QC H3G 1M8, Canada; sa_kalle@live.concordia.ca (S.K.); manar.amayri@concordia.ca (M.A.)

**Keywords:** interpretable machine learning, decision tree interpretability, electricity load profiling, clustering algorithms, cluster validation indices (CVIs), data characteristics, dimensionality reduction

## Abstract

Efficient energy management relies on uncovering meaningful consumption patterns from large-scale electricity load demand profiles. With the widespread adoption of sensor technologies such as smart meters and IoT-based monitoring systems, granular and real-time electricity usage data have become available, enabling deeper insights into consumption behaviors. Clustering is a widely used technique for this purpose, but previous studies have primarily focused on a limited set of algorithms, often treating clustering as a black-box approach without addressing interpretability. This study explores a wide number of clustering algorithms by comparing hard clustering algorithms (K-Means, K-Medoids) versus soft clustering techniques (Fuzzy C-Means, Gaussian Mixture Models) in segmenting electricity consumption profiles. The clustering performance is evaluated using five different clustering validation indices (CVIs), assessing intra-cluster cohesion and inter-cluster separation. The results show that soft clustering methods effectively capture inter-cluster characteristics, leading to better cluster separation, whereas intra-cluster characteristics exhibit similar behavior across all clustering approaches. This study assesses which CVIs provide reliable evaluations independent of clustering algorithm sensitivity. It provides a comprehensive analysis of the different CVIs’ responses to changes in data characteristics, highlighting which indices remain robust and which are more susceptible to variations in cluster structures. Beyond evaluating clustering effectiveness, this study enhances interpretability by introducing two decision tree models, axis-aligned and sparse oblique decision trees, to generate human-readable rules for cluster assignments. While the axis-aligned tree provides a complete explanation of all clusters, the sparse oblique tree offers simpler, more interpretable rules, emphasizing a trade-off between full interpretability and rule complexity. This structured evaluation provides a framework for balancing transparency and complexity in clustering explanations, offering valuable insights for utility providers, policymakers, and researchers aiming to optimize both clustering performance and explainability in sensor-driven energy demand analysis.

## 1. Introduction

With the global population growing and urbanization accelerating, energy demand has reached remarkable levels and continues to increase daily. The share of electricity in total energy consumption is expected to rise from 16% in 2002 to 20% by 2030, with global electricity consumption projected to double over the same period [1]. This growth is mainly driven by advancements in technology [2]. This highlights the need for efficient energy management strategies that can optimize consumption, reduce costs, and minimize environmental impact. Recent advancements in smart buildings and Internet of Things (IoT) sensor networks have introduced new opportunities for real-time energy monitoring and management. Smart meters, building automation systems, and energy sensors allow continuous tracking of electricity usage, providing large volumes of data that can be analyzed. The integration of machine learning and clustering techniques with these smart infrastructures enables data-driven optimization of energy consumption, improving energy efficiency and reducing peak demand. However, making sense of such vast, high-dimensional energy datasets remains a challenge, requiring effective data mining techniques to extract valuable insights. To address these challenges, data mining [3], also known as knowledge discovery from data [4], has emerged as a process for extracting insights from large and complex datasets. It involves the use of data analysis and discovery algorithms to identify specific patterns or models within the data [5]. Among its many techniques, clustering is the process of grouping objects with minimal or no prior knowledge of their relationships within the data. It aims to uncover underlying patterns or classes, grouping similar objects into the same cluster while ensuring they differ from objects in other clusters [6]. These clusters help identify consumption patterns, enabling customized energy-saving strategies, demand response programs, and better infrastructure planning. However, clustering energy data presents challenges such as high dimensionality, outliers, overlapping clusters, and varying densities, which can affect clustering accuracy and interpretability. Several studies have explored clustering techniques for energy data, but they often have limitations that affect their applicability. Some research focuses on interpretable clustering methods but fails to evaluate a broad set of clustering algorithms, lacks explainability mechanisms, or does not explore dimensionality reduction effects on clustering performance. Ref. [7] introduced an optimization-driven tree-based clustering model, which directly constructs tree-based clusters. While their method ensures inherent interpretability by structuring clusters as leaves in a decision tree, it does not compare traditional clustering methods such as K-Means, K-Medoids, Fuzzy C-Means, or Gaussian Mixture Models (GMM). Additionally, their study does not focus on clustering electricity consumption data, nor does it analyze how different decision tree models affect post-clustering explainability. In contrast, ref. [8] explored clustering interpretability but focused primarily on K-Means and K-Median, failing to evaluate soft clustering techniques like GMM or FCM. Other research, such as [9,10], applied clustering to electricity consumption data but only tested a limited number of clustering methods, restricting their ability to assess which techniques best segment real-world energy consumption patterns. Even studies that incorporated ensemble clustering techniques, such as [11], relied on a single clustering approach (GMM), without evaluating how different clustering techniques handle variations in electricity demand. Beyond the choice of clustering algorithms, another major limitation in prior research is the lack of explainability, which results in clustering being treated as a black-box method with no clear justification for why certain households belong to specific clusters. Studies such as [12,13] applied clustering to energy consumption data without incorporating post-clustering interpretability techniques, leaving energy analysts with limited insight into why clusters were formed. A recent survey on interpretable clustering [14] categorizes explainable clustering approaches into pre-clustering, in-clustering, and post-clustering techniques, reviewing how existing methods incorporate interpretability at different stages. However, this survey remains theoretical and does not provide an empirical comparison of clustering algorithms, limiting its ability to assess the practical effectiveness of explainability methods. While some studies attempted to integrate explainability using decision trees, such as [8], these efforts were often restricted to a single decision tree model, without evaluating how different tree structures impact interpretability. Ref. [7] introduced an optimization-driven tree-based clustering model, but it did not compare axis-aligned vs. sparse oblique decision trees, leaving a gap in understanding the trade-offs between full cluster coverage and rule simplicity. While some prior studies have investigated explainability in clustering [7,8,14], none have systematically compared multiple clustering methods on electricity consumption data while simultaneously evaluating different decision tree models for post-clustering interpretability. This study fills this gap by providing an empirical comparison of both clustering and explainability techniques, ensuring that results are interpretable and applicable to real-world energy planning. In addition to interpretability challenges, previous research often overlooked the impact of dimensionality reduction on clustering performance. Many studies apply dimensionality reduction techniques before clustering without testing how dimensionality reduction affects cluster quality. For example, ref. [10] used PCA but did not test whether clustering performance was improved or degraded by reducing feature dimensions. Similarly, ref. [11] performed clustering on high-dimensional electricity load data without testing whether reducing dimensionality could help improve segmentation accuracy. This gap is significant because dimensionality reduction may either enhance clustering efficiency or lead to information loss, depending on the dataset. Furthermore, the evaluation of clustering methods in prior studies was often limited to a single or multiple Cluster Validity Indices (CVIs), making it difficult to assess the robustness of the clustering results. While [10] provided a comprehensive analysis of multiple CVIs, most previous research relied solely on the silhouette score, Davies-Bouldin Index (DBI), or Dunn Index (DI), without testing their reliability under different data conditions. Since some CVIs are sensitive to specific data characteristics like density variations, skewness, and overlapping clusters, it is crucial to assess multiple CVIs to ensure that clustering evaluations are robust and not biased by a single metric.

### 1.1. Contributions of the Paper

This paper aims to fill the existing research gap by providing a comprehensive and interpretable clustering framework for electricity consumption profiling. Unlike previous studies that focused on a limited set of clustering methods or overlooked the role of dimensionality reduction, this study systematically evaluates both clustering performance and interpretability, ensuring a more robust and practical approach to electricity load segmentation. Furthermore, this study investigates the impact of data characteristics on clustering results by analyzing both inter-cluster (outliers, overlapping clusters, and density variations) and intra-cluster (skewness, kurtosis, and subclustering) properties. This enables a more informed selection of clustering algorithms and validation metrics, ensuring accurate and reliable clustering evaluations. The main contribution of this study is the integration of interpretability into clustering analysis through decision-tree-based explainability techniques. Unlike previous works where clustering results were treated as a black-box, this study employs axis-aligned and sparse oblique decision trees to extract human-readable decision rules, making clustering results more transparent, explainable, and actionable for energy policymakers, utility providers, and analysts. This contribution ensures that consumers’ energy consumption patterns are not only identified but also understood in a way that facilitates informed decision making. The key contributions of this paper can be summarized as follows:

- Clustering interpretability through decision trees;
- Comprehensive clustering comparison;
- Impact of dimensionality reduction on clustering performance;
- Evaluation of five Cluster Validity Indices (CVIs) for reliable assessment.

### 1.2. Outline of the Paper

The paper first presents the methodology framework and materials used in this study in Section 2. This is followed by a detailed presentation of the results along with corresponding discussions (Section 3). Section 4 focuses on the interpretability analysis, providing insights into the explainability of clustering results using decision trees. Finally, the paper concludes in Section 5, summarizing the key findings and outlining potential directions for future research.

## 2. Materials and Methods

**Figure 1:**
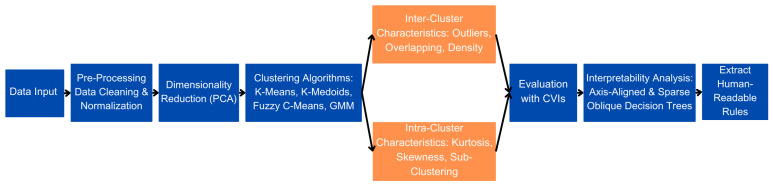
Methodological framework outlining data preprocessing, clustering, inter-/intra-cluster evaluation, and interpretability analysis.

To analyze electricity consumption patterns and enhance clustering interpretability, this study follows a structured methodology that integrates data pre-processing, clustering, cluster validation, and explainability techniques. Figure 1 provides an overview of the complete workflow, outlining the key steps involved in the analysis.

### 2.1. Data and Pre-Processing

**Figure 2:**
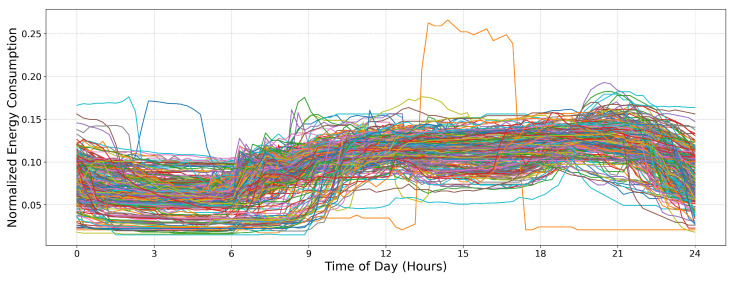
Comparison of representative daily profiles across 315 households in the original feature space. Each curve represents a single household’s normalized electricity consumption over a 24 h period.

This study analyzes electric load demand profiles using a publicly available dataset from the Electricity Load Diagram (EL) Dataset, part of the UCI Machine Learning Repository [15]. The dataset contains electricity consumption data recorded at 15 min intervals over a four-year period (2011–2014). As smart buildings and connected energy systems become more common, such datasets provide insights into electricity usage, facilitating demand prediction, anomaly detection, and energy efficiency improvements. Initially, data were collected from 370 Portuguese consumers, but due to late participation and missing records, a pre-filtering process was applied. Only households with at least 900 days of complete data were retained, reducing the sample size to 315 households. To ensure data consistency, specific days were excluded from the dataset. The last Sundays of March and October were removed since they coincide with daylight-saving time changes in Portugal and the U.S., potentially introducing inconsistencies in consumption patterns. Since the main objective of this study is to cluster daily load demand profiles rather than the data themselves, removing these days was considered appropriate. Rather than using raw daily records, which may contain seasonal fluctuations or temporary anomalies, a representative daily profile was computed for each household. This was achieved by calculating the median consumption value for each time slot across all available days, ensuring that only valid and complete daily profiles were considered. Using the median instead of the mean prevents outliers (e.g., extreme consumption days) from distorting the profile, making clustering results more stable. To enable meaningful comparisons across households, L2 normalization was applied to the median daily profiles. This transformation scales each household’s profile vector to have a unit norm, ensuring that the clustering process emphasizes the shape and distribution of consumption patterns rather than absolute electricity usage levels. The L2 normalization is defined as:

$$X_{\mathrm{normalized}}=\frac{X}{{\left||X|\right|}_{2}}=\frac{X}{\sqrt{\sum_{i=1}^{d}X_{i}^{2}}}$$

where *X* represents the original daily profile vector containing multiple time slots for each day and ${\left||X|\right|}_{2}$ is the L2 norm. The resulting vector, $X_{\mathrm{normalized}}$, is the final scaled profile used for clustering, ensuring that variations in total consumption do not affect the identification of structural patterns in the data. To visualize the daily consumption patterns across all households, Figure 2 presents a comparison of 315 representative daily profiles in the original feature space. Each curve corresponds to a single household, showcasing the variability in energy usage over 24 h. The overlapping patterns reveal common consumption trends, such as morning and evening peaks, while also highlighting individual differences in household energy behavior. This visualization provides an overview of how normalized load demand profiles differ across consumers, reinforcing the need for clustering to identify distinct consumption patterns.

### 2.2. Dimensionality Reduction

Each consumer’s electric load demand profile is structured as a time-series vector with 96 distinct values, where each value represents electricity consumption over a 15 min interval within a 24 h period. Since there are four intervals per hour, the total number of dimensions is computed as $24\times \frac{60}{15}=96$. This vectorized representation effectively captures each consumer’s daily consumption pattern. However, high-dimensional data present challenges, particularly the curse of dimensionality, which lowers the performance of clustering algorithms by introducing noise and increasing computational complexity [16].To address these challenges, Principal Component Analysis (PCA) [17], a widely used dimensionality reduction technique, was applied. To ensure interpretability, a feature mapping approach was employed, where the contribution of each original feature to the principal components was analyzed. This was achieved by examining the PCA loadings, which indicate how strongly each 15 min interval influences a given principal component. The top contributing features for each principal component were identified and stored in a structured table to establish a meaningful connection between the reduced dimensions and the original electricity consumption patterns. The dimensionality reduction technique was applied to the EL dataset after standardizing the data. Standardization is essential to ensure that all features contribute equally to the analysis. The main goal of applying PCA is to reduce the number of dimensions while keeping the most significant variance within the data. For this purpose, the optimal number of dimensions needs to be identified. The optimal number of principal components was determined using the Cumulative Explained Variance Ratio (CEVR) method. It identifies the number of components required to capture a substantial portion of the total variance [18]. To further validate the optimal number selected, incremental variance analysis was conducted. This method works by assessing the additional variance explained by each subsequent component until the incremental gain falls below a pre-defined threshold. The two combined methods ensure an accurate determination of the number of components to consider and enhance the efficiency of dimensionality reduction process. Applying PCA could potentially enhance the efficiency and interpretability of subsequent clustering analysis.

### 2.3. Clustering Algorithms

With the challenges of high-dimensional data now reduced, the next step is to use clustering algorithms to uncover patterns within customer profiles. Four clustering algorithms were applied to analyze customer consumption patterns—K-Means, K-Medoids, Fuzzy C-Means, and Gaussian Mixture Models (GMM)—to the EL dataset with and without dimensionality reduction. Unlike the previous study, which primarily relied on K-Means for the EL dataset with dimensionality reduction and Fuzzy C-Means on another dataset with dimensionality reduction only, this study tests a broader range of algorithms.

Each of these algorithms was chosen based on its ability to address specific challenges in clustering electricity load demand profiles: K-Means is one of the most popular clustering algorithms. It was selected as a baseline due to its efficiency and widespread use in clustering time-series data [19]. It is known as a hard clustering algorithm [20], meaning it assigns each data point exclusively to one cluster without any shared membership across clusters. This method partitions data into distinct groups by minimizing the sum of squared distances between data points and their respective cluster centroids. However, K-Means is sensitive to outliers because an extreme value heavily influences the mean, leading to suboptimal clustering results [21]. To address this challenge, K-Medoids was employed, which is another hard clustering algorithm. However, it considers cluster centers (medoids) instead of the mean, which is not affected by extreme values. It assigns each data point to the nearest medoid based on a specified distance metric [22]; in this research, Euclidean distance was chosen. Using the medoid reduces the impact of outliers on the clustering structure [23].

Knowing that hard clustering methods may not effectively capture clusters with outliers and overlapping characteristics, soft clustering [20] techniques were adopted to improve the clustering analysis. In soft clustering, data points can belong to multiple clusters with varying probabilities. Fuzzy C-Means [24] is a soft clustering algorithm that assigns each data point a degree of membership to different clusters. The membership value is calculated based on the distance between the data point and randomly chosen centers. Higher membership is associated with points that are closer to a cluster center, while other distant points receive lower values. This approach helps to identify clusters with overlapping and shared features. To further validate our assumptions about soft clustering, GMM [25] were applied.

To apply the clustering algorithms discussed, the optimal number of clusters needs to be identified as a first step. For K-Means, the optimal number of clusters was determined using both the elbow heuristic [26] method and the gap statistic [27] method. The elbow method calculates the sum of squared distances from each data point to its assigned cluster centroid. This metric, known as the within-cluster sum of squares (WSS), measures the compactness of clusters, with lower values indicating tighter, more well-defined groupings. When comparing datasets with different dimensionalities, it is more appropriate to use the average WSS, which normalizes the sum of squared distances by the number of data points. This approach makes the comparison fair by reducing the impact of higher-dimensional spaces, where distances naturally increase. As a result, it provides a more accurate way to assess clustering quality. The optimal number of clusters is the point that balances the sum of squared distances and model complexity. Essentially, it is the point where adding another cluster results in only a marginal improvement in variance reduction. The second method is the gap statistic, which determines the optimal number of clusters by comparing the clustering quality of the actual dataset to that of a randomly distributed reference dataset. It evaluates how much more compact the clusters in the real data are compared to randomly scattered points, ensuring that the chosen number of clusters reflects meaningful structure rather than random variations. The gap statistic is computed by first running clustering on the real dataset and calculating WSS. A random dataset with the same size and range is then generated, and clustering is applied to obtain its WSS. The gap statistic is defined as:

$$\mathrm{Gap}(k)=E^{*}\left[\log(WSS_{k})\right]-\log(WSS_{k})$$

where $WSS_{k}$ is the within-cluster sum of squares for *k* clusters in the real dataset and $E^{*}\left[\log(WSS_{k})\right]$ is the expected log WSS for the random dataset. A larger gap indicates that the real data exhibits stronger clustering structure. The ideal number of clusters is where the gap between real and random clustering is largest [27].

For K-Medoids, gap statistic was used to determine the optimal number of clusters. For Fuzzy C-Means, the Dunn Index [28] was used to select the optimal number of clusters. The Dunn Index calculates the ratio of the minimum inter-cluster distance to the maximum intra-cluster distance. Finally, for GMM, the silhouette score [28] was employed, which calculates the average distance between points within the same cluster compared to points in neighboring clusters.

### 2.4. Intra-Cluster and Inter-Cluster Analysis

To analyze clustering performance, the next step is to examine structural properties within and between clusters to identify influencing factors. By examining inter-cluster characteristics which are outlier, overlapping, and density, and intra-cluster characteristics which are skewness, kurtosis, and subclustering, adjustment could be done to enhance cluster quality. This study evaluates the impact of data characteristics on clusters using CVIs.

#### 2.4.1. Inter-Cluster Analysis

Inter-cluster characteristics define relationships between clusters. In this study, outlier, overlapping, and differential density are analyzed.

- Outliers are defined as single-point clusters in the clustering results. These points are included in the clustering process but are not expected to significantly influence the CVI scores. To verify this assumption, outliers were identified through manual inspection and subsequently removed. CVI scores were then compared before and after the removal of these single-point clusters to evaluate any potential impact on the clustering validation metrics.
- Overlapping clusters occur when certain data points share characteristics with more than one cluster, making it difficult to assign them definitively to a single cluster. This typically arises when clusters are not well-separated, with some data points located near the boundaries of two or more clusters. To address this issue, overlapping points were removed from the dataset to evaluate whether reducing overlap could improve cluster quality. For Fuzzy C-Means without and with DR, GMM without DR, and K-Means without DR, overlapping points were identified and removed through manual inspection. For GMM with DR, K-Medoids with and without DR, and K-Means with DR, a distance-based approach was applied. This method calculates the Euclidean distance between each data point and the cluster centers. Points found to be close to multiple centers within a specified threshold were considered overlapping and removed. The threshold was manually adjusted to balance effectiveness in reducing overlap while maintaining data integrity.
- Differential density refers to clusters having varying densities, often reflecting natural consumption patterns. Density is defined as the number of data points per unit volume and was calculated as the ratio of points in a cluster to its diameter. It was hypothesized that increasing the density of clusters would improve CVI scores. To evaluate this, the density of the densest cluster was increased by adding additional points while keeping the cluster diameter constant. The effect of this adjustment on CVI scores was then analyzed to assess the relationship between cluster density and clustering quality.

#### 2.4.2. Intra-Cluster Analysis

Intra-cluster characteristics refer to the internal structural properties of clusters. In this study, these characteristics are assessed through the analysis of kurtosis, skewness, and subclustering.

- Central kurtosis refers to data points being tightly clustered around the center of a cluster, with fewer points near the edges. Statistically, kurtosis measures the peakedness or flatness of a distribution. When more points are concentrated near the center, the distribution becomes leptokurtic, increasing similarity within the cluster. This increased density is expected to enhance CVI scores by making clusters more compact. To evaluate this, *k*% of the data points in each cluster were shifted closer to the center, where *k* ∈ {25, 50, 75, 100}. The shifted points were generated on a *d*-dimensional hypersphere, where *d* corresponds to the data dimensionality. The radius of the hypersphere was set to the cluster radius divided by *m*, where *m* is a discrete value ranging from 5 to 20, depending on the dimensionality of the data. The effect of these adjustments on CVI scores was analyzed to assess the impact of central kurtosis on clustering performance.
- Skewness occurs when the mean is positioned away from the cluster’s center, resulting in an asymmetrical distribution. While kurtosis increases density near the center, skewness shifts the majority of data points toward one side of the cluster, causing the mean to deviate from the geometric center. With the cluster’s diameter remaining constant, increasing skewness is expected to either maintain or improve CVI scores. To evaluate this, *k*% of the data points in each cluster were rearranged farther from the center and closer to the mean, where *k* ∈ {25, 50, 75, 100}. To preserve the natural structure of each cluster, no new points were added to empty regions. Adjustments were performed around the mean to introduce skewness without creating artificial patterns inconsistent with real-world data distributions. During implementation, the distance between the mean and the center was calculated for all clusters. If this distance exceeded the cluster radius divided by *n*, skewness adjustments were applied, where *n* is a discrete value ranging from 2 to 5, depending on the cluster size. The rearranged points were positioned on a *d*-dimensional hypersphere, where *d* corresponds to the data dimensionality. The radius of the hypersphere was set to the cluster radius divided by *m*, where *m* is a discrete value ranging from 5 to 20, based on the data dimensionality.
- Subclustering occurs when a cluster splits into two or more smaller clusters. This suggests that the cluster structure is not optimal and that dividing it into smaller, distinct clusters may yield better results. Such a division indicates that the subclusters do not share significant characteristics or features, warranting their distinction as separate clusters. The presence of subclusters is expected to worsen CVI scores due to reduced cohesion and separation within the clusters. To evaluate the impact of subclustering, the mean and center of each cluster were analyzed, as these are considered dense points. The distance between the mean and the center of each cluster was calculated and compared to a threshold defined for each algorithm. If this distance exceeded the threshold, subclustering was identified. In such cases, points were rearranged to move closer to the nearest dense point (mean or center). These rearrangements were performed with adjustments affecting {25, 50, 75, 100} of the points within the cluster.

### 2.5. Cluster Validity Indices

Building upon the structural analysis of clusters, CVIs were employed to quantitatively assess clustering performance. They provide metrics to evaluate aspects such as cohesion, separation [29], and compactness [29,30] of clusters. In this study, five clustering validation indices were used: silhouette score (SH), Calinski–Harabasz Index (CH), Dunn Index (DI), Davies–Bouldin Index (DB), and Xie–Beni Index (XB).

Silhouette Score (SH): This index measures the quality of clustering by calculating the ratio of cohesion (similarity within clusters) to separation (difference between clusters). Scores range from −1 to 1, with higher values indicating well-defined clusters [31]. The SH score was chosen for its straightforward interpretability and its ability to provide a normalized measure of clustering performance that is independent of the number of clusters.

Calinski–Harabasz (CH) Index: Also known as the Variance Ratio Criterion, this index evaluates the ratio of between-cluster dispersion to within-cluster dispersion. Higher scores suggest compact, well-separated clusters [32]. The CH index was selected because it effectively balances compactness and separation, making it particularly suitable for evaluating clustering outcomes in datasets with varying cluster densities.

Davies–Bouldin (DB) Index: This index assesses clustering quality based on the average similarity ratio between each cluster and its most similar counterpart. Lower DB values indicate better-defined clusters with greater separation [30]. The DB index was included because it measures how well clusters are separated and how compact they are, making it useful even when clusters overlap or are not perfectly shaped.

Dunn’s (DI) Index: This index identifies compact and well-separated clusters by maximizing the minimum inter-cluster distance and minimizing the maximum intra-cluster distance. Higher values indicate superior clustering performance [33]. The DI index was chosen for its robustness in identifying well-separated clusters, particularly in datasets where compactness and separation are critical for evaluation.

Xie–Beni (XB) Index: Commonly used in fuzzy clustering, this index compares within-cluster scatter to between-cluster separation. Lower XB values reflect better clustering quality [29]. The XB index was selected for its sensitivity to variations in cluster compactness and separation, providing a reliable measure.

## 3. Results and Discussion

### 3.1. Principal Component Analysis

**Figure 3:**
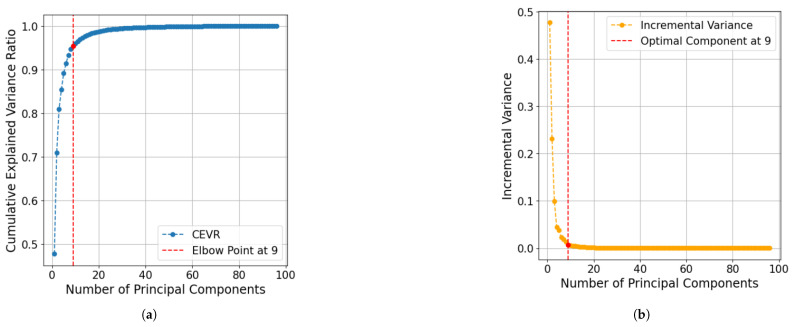
(**a**). Plot of the number of principal components versus CEVR. Elbow point is the optimal number of reduced dimensions while performing the PCA. (**b**). Plot of the number of principal components versus incremental variance. Optimal component is the optimal number of reduced dimensions while performing PCA.

PCA served as an important step in simplifying the dataset while retaining its most significant features. Figure 3 illustrates the Cumulative Explained Variance Ratio (CEVR) across the principal components, with an elbow point at the ninth component. This point captures 95% of the total variance. Any additional components beyond the elbow point yield minimal variance, indicating diminishing returns. To further validate the selection of nine components, Figure 3 showcases an incremental variance analysis. This analysis confirms that components beyond the ninth contribute less than a practical threshold of 0.01 variance. This finding reinforces that nine components represent the optimal number to retain in this study. Since a dimensionality reduction technique was applied to the EL dataset, a feature mapping was generated during PCA to link each principal component to the original features. This mapping facilitates the interpretation of decision rules in Section 4 and is presented in Table 1.

### 3.2. Clustering Algorithms

**Figure 4:**
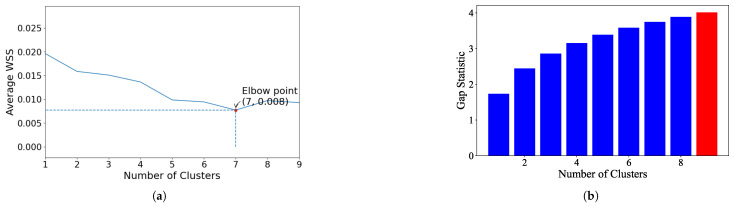
(**a**). Plot of the number of clusters versus average within sum of squares. Elbow point is the optimal number of clusters while performing K-Means on EL without dimensionality reduction. (**b**). Value of the gap statistic for different number of clusters to identify the optimal number of clusters for the K-Means algorithm on EL without dimensionality reduction. Optimal value of gap statistic is highlighted in red.

**Figure 5:**
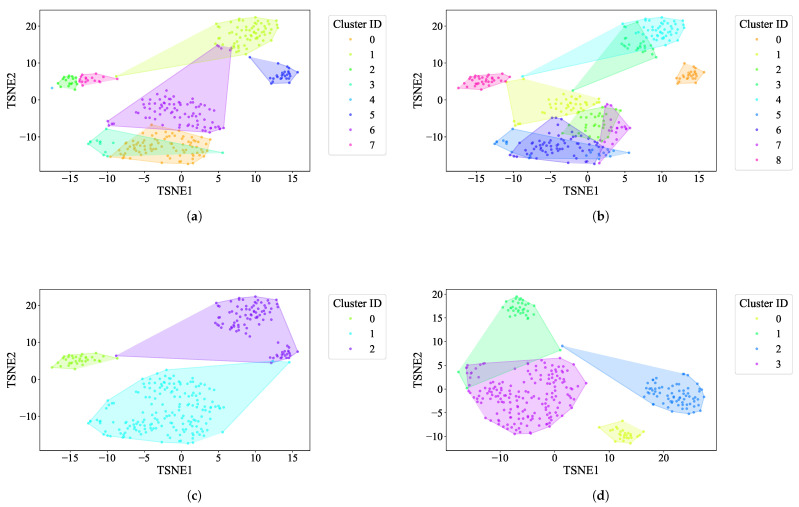
The t-SNE plot illustrates baseline clustering results using different algorithms on the EL dataset without dimensionality reduction: (**a**) K-Means, (**b**) K-Medoids, (**c**) Fuzzy C-Means, and (**d**) GMM.

**Figure 6:**
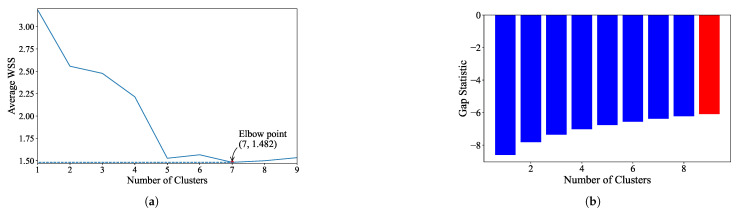
(**a**). Plot of the number of clusters versus CEVR. Elbow point is the optimal number of clusters while performing K-Means on EL with dimensionality reduction. (**b**). Value of the gap statistic for different number of clusters to identify the optimal number of clusters for the K-Means algorithm on EL with dimensionality reduction. The optimal value of the gap statistic is highlighted in red.

**Figure 7:**
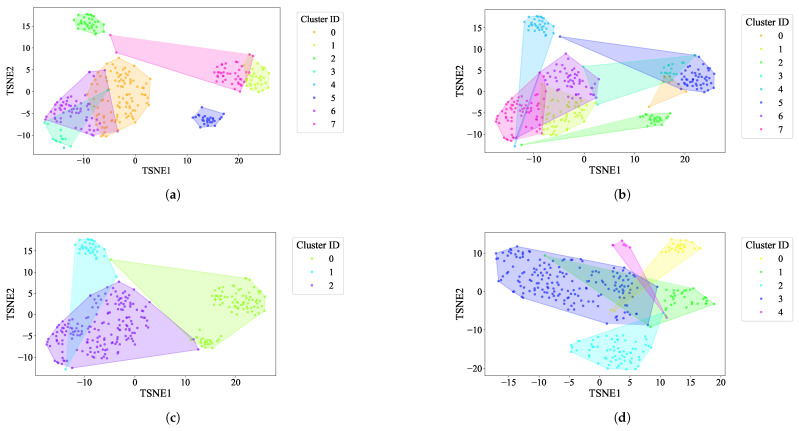
The t-SNE plot illustrates baseline clustering results using different algorithms on the EL dataset with dimensionality reduction: (**a**) K-Means, (**b**) K-Medoids, (**c**) Fuzzy C-Means, and (**d**) GMM.

**Figure 8:**
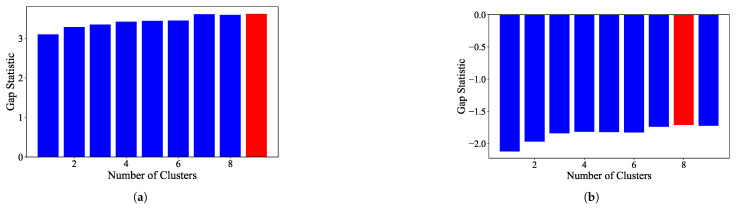
Value of the gap statistic for different numbers of clusters to identify the optimal number of clusters for the K medoids algorithm on EL. Optimal value of the gap statistic is highlighted in red. (**a**). EL dataset without dimensionality reduction (**b**). EL dataset with dimensionality reduction.

- K-Means: For the EL dataset without DR, the optimal number of clusters was determined using two methods. The elbow heuristic suggested seven clusters while the gap statistic recommended nine clusters, as shown in Figure 4. To correct this difference, eight clusters were selected as the optimal number. Applying K-Means with eight clusters produced the visualization shown in Figure 5, which reveals overlapping clusters and the presence of an outlier. For the EL dataset with DR, the same process was followed. The elbow heuristic again suggested seven clusters while the gap statistic indicated nine clusters, as presented in Figure 6. Based on both methods, eight clusters were again selected as the optimal number. The clustering results, displayed in Figure 7, also show overlapping clusters and the presence of an outlier in cluster 4. K-Means, as a hard clustering algorithm, is known to be sensitive to outliers. This sensitivity is evident in the clustering results presented, where outliers were present alongside overlapping clusters. The reliance on the mean as the cluster center causes outliers to significantly influence the cluster boundaries, resulting in less accurate cluster definitions.
- K-Medoids: To address the identified outlier issue, the K-Medoids algorithm was applied. For the EL dataset without DR, the gap statistic suggested an optimal number of clusters equal to nine while for EL dataset with DR, the gap statistics proposed eight clusters (see Figure 8). After applying K-Medoids clustering with nine clusters and with eight clusters, the results, shown in Figure 5 and Figure 7, respectively, demonstrated effective handling of the outlier. However, overlapping still exists in both cases. K-Medoids, as a hard clustering algorithm, solved the outlier issue. The removal of outliers demonstrates its effectiveness in correcting this limitation of K-Means. However, overlapping clusters persisted, indicating that while K-Medoids can handle outliers, it does not correctly address the challenge of overlapping. This underscores a limitation of hard clustering methods, as they rely on strict boundaries that cannot account for shared characteristics between clusters.
- Fuzzy C-Means: To address the issue of overlapping clusters, the soft clustering algorithm Fuzzy C-Means was applied. For the EL dataset without DR, the Dunn Index identified the optimal number of clusters as 3, with a fuzzifier value of 1.2. The clustering results, presented in Figure 5, show that Fuzzy C-Means minimized overlapping compared to hard clustering techniques. For the EL dataset with DR, the Dunn Index similarly indicated an optimal cluster count of 3, with a slightly adjusted fuzzifier value of 1.1. The clustering results, displayed in Figure 7, show no outliers and minimal overlapping, although the overlap was slightly greater than in the non-DR case.
  Fuzzy C-Means resulted in a marked reduction in overlap. The probabilistic nature allows it to handle data points that fall near cluster boundaries. This results in smoother transitions between clusters and a better representation of complex data structures. Furthermore, no outliers were observed, which highlight its robustness. Dimensionality reduction had no noticeable effect on these results, as similar improvements were observed for both datasets with and without DR.
- Gaussian Mixture Model: The second soft clustering algorithm applied was the GMM, where the silhouette score was used to determine the optimal number of clusters. For the EL dataset without DR, the SH identified four as the optimal number of clusters. The clustering results, presented in Figure 5, show well-defined clusters with minimal overlap and no outliers, demonstrating the capability of GMM to handle overlapping clusters effectively. For the EL dataset with DR, SH indicated five as the optimal number of clusters, as shown in Figure 7. The clustering results reveal no outliers but show a slightly higher degree of overlap compared to the dataset without DR. The presented results further validate the advantages of soft clustering. Like Fuzzy C-Means, results from GMM demonstrated reduced overlap compared to hard clustering methods and the complete absence of outliers. GMM’s ability to model the data probabilistically improves its flexibility in representing real-world data, particularly in high-dimensional spaces. Dimensionality reduction similarly showed no significant impact on the results, as the improvements in clustering quality were consistent across both datasets.
  Findings from both the EL datasets, with and without dimensionality reduction, demonstrated that soft clustering is better than hard clustering in terms of outlier and overlapping. While K-Means and K-Medoids struggle with overlapping clusters, soft clustering algorithms, Fuzzy C-Means, and GMM effectively reduce overlap and eliminate outliers. This reinforces the strength of soft clustering approaches in handling complex data distributions, where clusters may share characteristics or where outliers significantly impact results. The absence of a noticeable effect from dimensionality reduction suggests that the algorithms themselves played a more critical role in improving clustering quality than the pre-processing step.

**Figure 9:**
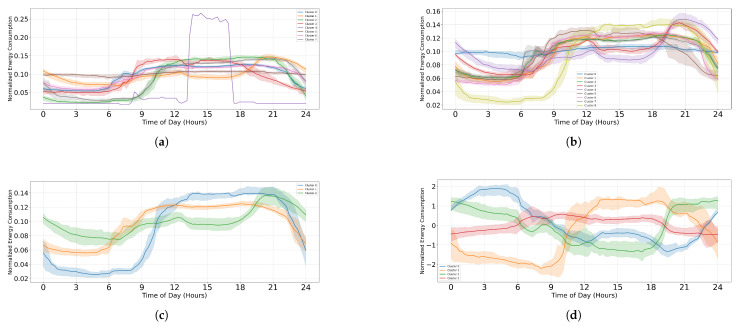
Comparison of representative daily profiles across clusters using the EL dataset without dimensionality reduction. (**a**) K-Means, (**b**) K-Medoids, (**c**) Fuzzy C-Means, and (**d**) GMM.

To further explore the characteristics of each cluster, representative daily energy consumption profiles were visualized for each clustering algorithm. Figure 9 offers insight into how energy usage varies across different clusters throughout the day, revealing general trends such as morning and evening peaks or fluctuations in consumption.

Figure 9 highlights key differences in clustering behavior across algorithms. In K-Means (see Figure 9a), the presence of an outlier is evident, as represented by the purple cluster, which exhibits an unusual spike around midday, a characteristic not observed in the other clustering methods. Additionally, overlapping between clusters is noticeable, particularly in K-Means (see Figure 9a) and K-Medoids (see Figure 9b), where multiple clusters share similar consumption patterns, leading to ambiguity in boundary definitions. In contrast, Fuzzy C-Means (see Figure 9c) and GMM (see Figure 9d) exhibit more distinct and structured cluster separations. However, while these visualizations provide a high-level overview of cluster behavior, the underlying rationale behind cluster assignments remains a black box. There is no explicit understanding of the factors driving these groupings or the specific features that most strongly influence cluster formation. To bridge this interpretability gap, a combination of axis-aligned and sparse oblique decision trees is employed to extract meaningful classification rules, offering a transparent and structured way to understand and justify the clustering process.

### 3.3. Inter- and Intra-Cluster Characteristics

#### 3.3.1. Inter-Cluster Characteristics

**Figure 10:**
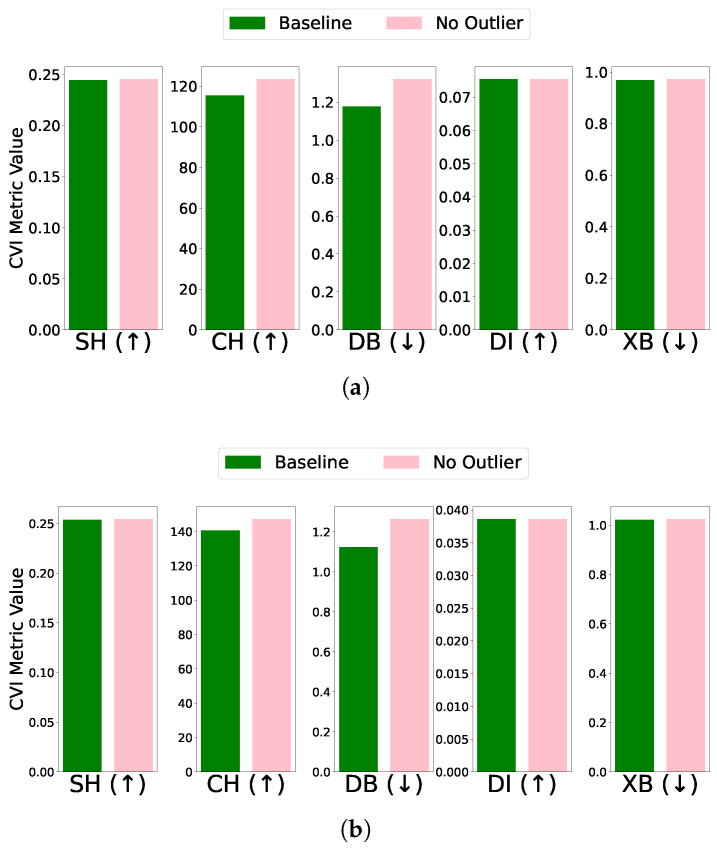
CVIs’ response to outlier removal using K-Means on the EL dataset. (**a**). EL dataset without applying dimensionality reduction. (**b**). EL dataset with dimensionality reduction.

**Figure 11:**
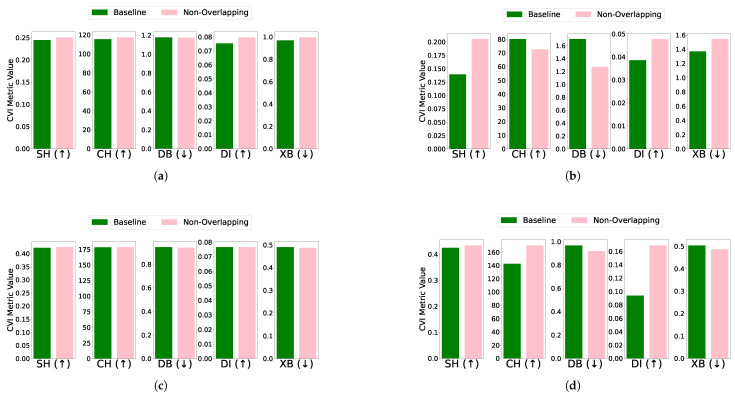
CVIs’ response to overlapping removal using different algorithms on the EL dataset without dimensionality reduction: (**a**) K-Means, (**b**) K-Medoids, (**c**) Fuzzy C-Means, and (**d**) GMM.

**Figure 12:**
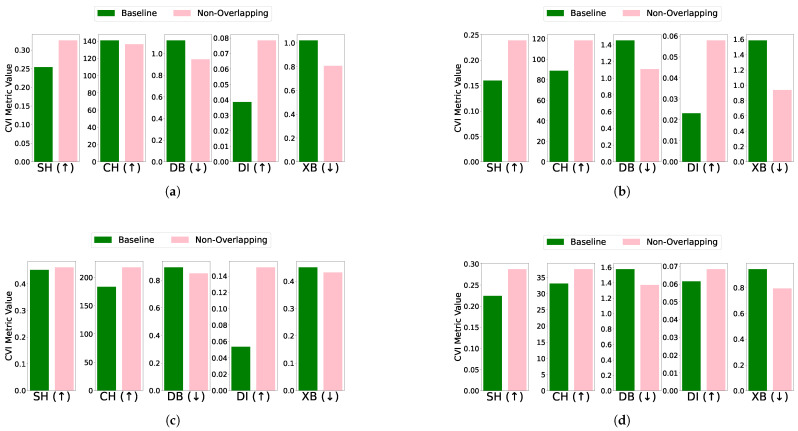
CVIs’ response to overlapping removal using different algorithms on the EL dataset with dimensionality reduction: (**a**) K-Means, (**b**) K-Medoids, (**c**) Fuzzy C-Means, and (**d**) GMM.

**Figure 13:**
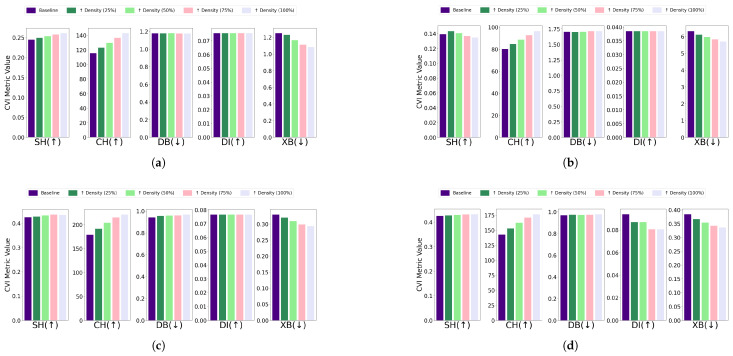
The effect of increasing differential density on various CVIs using different algorithms on the EL dataset without dimensionality reduction: (**a**) K-Means, (**b**) K-Medoids, (**c**) Fuzzy C-Means, and (**d**) GMM.

**Figure 14:**
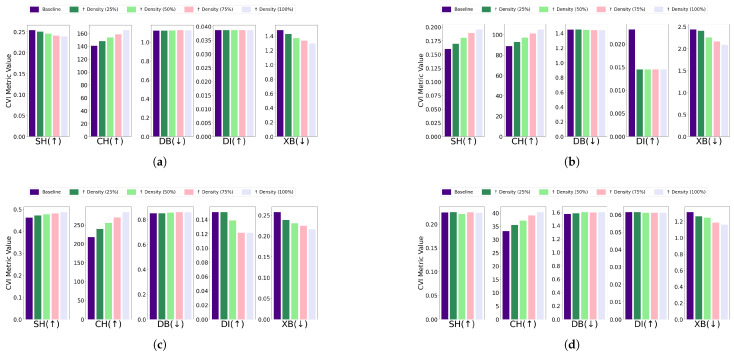
The effect of increasing differential density on various CVIs using different algorithms on the EL dataset with dimensionality reduction: (**a**). K-Means, (**b**), K-Medoids, (**c**). Fuzzy C-Means, and (**d**). GMM.

- Outlier: The impact of outlier removal on clustering performance was assessed by comparing CVI metrics for the baseline and outlier-removed cases. Among the algorithms, only K-Means identified outliers. In both the EL dataset without and with DR, SH, DI, and XB metrics remained unchanged after outlier removal. However, the CH score improved, while the DB index deteriorated unexpectedly in both cases (see Figure 10). Most CVIs remained unaffected, except for CH and DB. CH improved due to enhanced inter-cluster dispersion, as outlier removal increased the separation between clusters. Conversely, DB deteriorated unexpectedly, likely because the reduced intra-cluster variance was accompanied by diminished inter-cluster separation. This behavior suggests that SH, XB, or DI are reliable CVIs for datasets with outliers, while DB and CH require careful consideration due to their sensitivity to outlier removal.

- Overlapping: The effect of removing overlapping profiles on clustering performance was evaluated for each algorithm and dataset combination. For the EL dataset without DR, removing overlapping profiles improved clustering performance across algorithms. Specifically, K-Means showed improved CVI metrics, except for the XB index, which worsened; K-Medoids demonstrated enhancements in SH, DB, and DI indices, while CH and XB indices remained unchanged; Fuzzy C-Means exhibited minimal impact on CVIs after the removal of one profile; and GMM showed improvements across all CVIs after removing two profiles. For the EL dataset with DR, the removal of overlapping profiles resulted in more distinct clusters and noticeable improvements across all algorithms. K-Means exhibited substantial improvements in SH, DB, DI, and XB indices, with a slight deterioration in CH; K-Medoids and Fuzzy C-Means showed significant improvements across all CVIs; and GMM achieved better-defined clusters with enhanced CVI performance. The results are summarized in Figure 11 (without DR) and Figure 12 (with DR).
  Removing overlapping data points improved all CVIs across algorithms, both with and without DR, except for XB in K-Medoids without DR. This inconsistency in XB’s behavior highlights the need for further investigation, as it demonstrated improvements when DR was applied. For overlapping datasets, SH, CH, DI, and DB are recommended, while XB should be used cautiously, particularly in non-DR scenarios.
- Differential density: The impact of differential density on CVI metrics varied across clustering algorithms and dataset configurations. Without DR, most algorithms showed improvements in SH, CH, and XB indices, while DB and DI generally remained constant. Notably, GMM exhibited slight decreases in DI despite improvements in CH and XB. With DR, CH and XB consistently improved across all algorithms. However, K-Means and Fuzzy C-Means showed unexpected deterioration in SH, and K-Medoids and Fuzzy C-Means experienced decreases in DI. Full details are visualized in Figure 13 (without DR) and Figure 14 (with DR). Increasing the density of clusters improved most CVIs, except for DI and DB, which remained stable across all algorithms. This stability can be attributed to DI’s reliance on minimum inter-cluster distances, which are unaffected by density changes, and DB’s focus on the average similarity between clusters, which does not capture intra-cluster density variations. For datasets with increasing density, SH, XB, and CH are the most reliable CVIs, while DI and DB may be less informative.

#### 3.3.2. Intra-Cluster Characteristics

**Figure 15:**
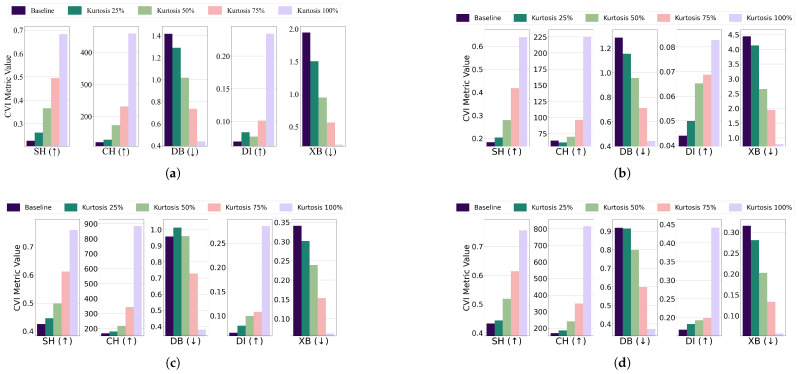
The effect of increasing the level of kurtosis close to center on various CVIs using different algorithms on the EL dataset without dimensionality reduction: (**a**) K-Means, (**b**) K-Medoids, (**c**) Fuzzy C-Means, and (**d**) GMM.

**Figure 16:**
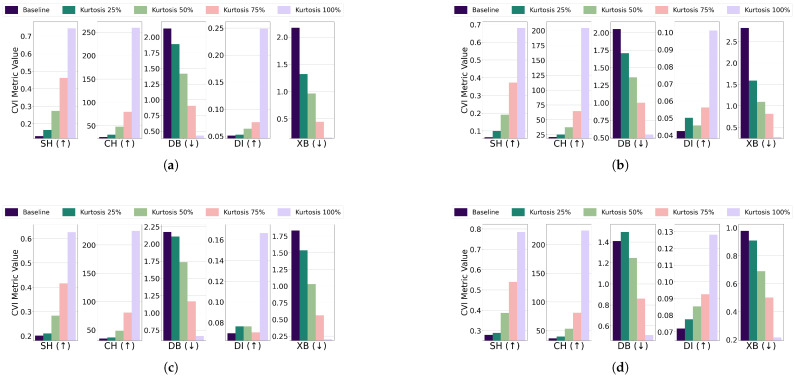
The effect of increasing the level of kurtosis close to center on various CVIs using different algorithms on the EL dataset with dimensionality reduction: (**a**) K-Means, (**b**) K-Medoids, (**c**) Fuzzy C-Means, and (**d**) GMM.

**Figure 17:**
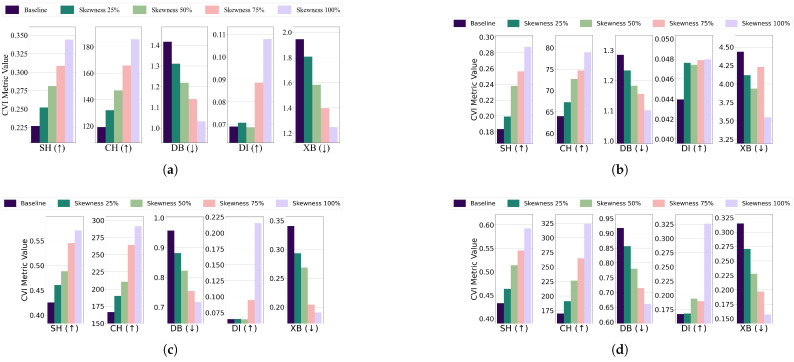
The effect of increasing the level of skewness on various CVIs using different algorithms on the EL dataset without dimensionality reduction: (**a**) K-Means, (**b**) K-Medoids, (**c**) Fuzzy C-Means, and (**d**) GMM.

**Figure 18:**
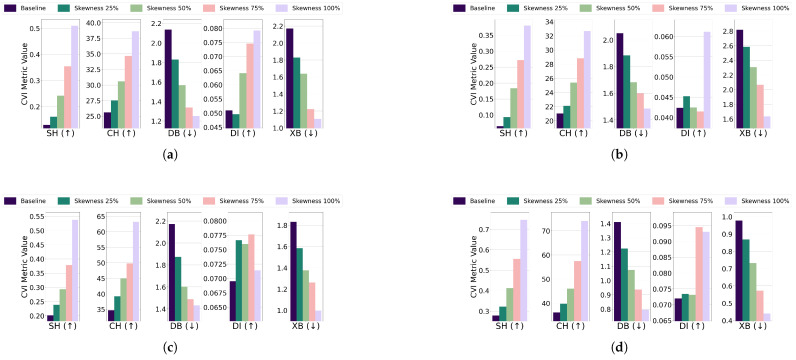
The effect of increasing the level of skewness on various CVIs using different algorithms on the EL dataset with dimensionality reduction: (**a**) K-Means, (**b**) K-Medoids, (**c**) Fuzzy C-Means, and (**d**) GMM.

**Figure 19:**
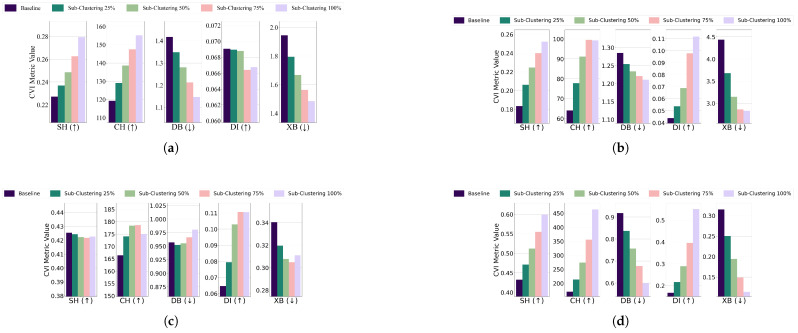
The effect of increasing the level of subclustering on various CVIs using different algorithms on the EL dataset without dimensionality reduction: (**a**) K-Means, (**b**) K-Medoids, (**c**) Fuzzy C-Means, and (**d**) GMM.

**Figure 20:**
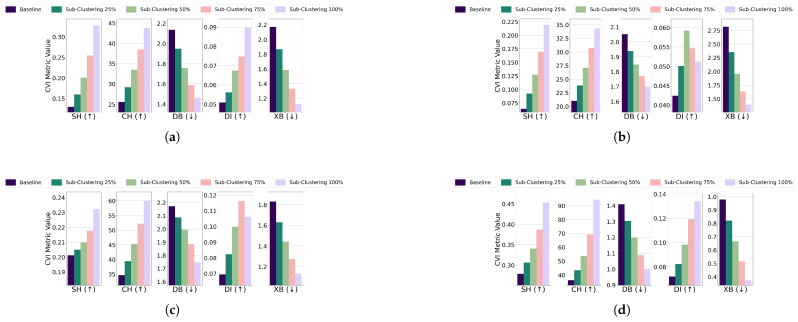
The effect of increasing the level of subclustering on various CVIs using different algorithms on the EL dataset with dimensionality reduction: (**a**) K-Means, (**b**) K-Medoids, (**c**) Fuzzy C-Means, and (**d**) GMM.

- Central kurtosis: The effect of increasing central kurtosis on clustering performance was evaluated for each algorithm and dataset combination. For the EL dataset without DR, points were adjusted to lie on a 96-dimensional hypersphere with a radius equal to $\frac{1}{8}$ of the cluster size. For the EL dataset with DR, points were shifted to a nine-dimensional hypersphere with a radius equal to $\frac{1}{15}$ of the cluster size. Across all algorithms—K-Means, K-Medoids, Fuzzy C-Means, and GMM—increasing central kurtosis led to improvements in CVI metrics for both versions of the EL dataset. The results for CVIs on the EL dataset without DR are displayed in Figure 15 while the results for the EL dataset with DR are shown in Figure 16. Increasing central kurtosis positively impacted all CVIs, as redistributing points closer to cluster centers enhanced both intra-cluster compactness and inter-cluster separation. This consistent improvement across algorithms suggests that SH, XB, CH, DI, and DB are all effective for datasets with central kurtosis.
- Skewness: For the EL dataset without DR, points were repositioned on a 96-dimensional hypersphere with a radius equal to $\frac{1}{5}$ of the cluster size. Adjustments led to improvements in CVI metrics across all algorithms. K-Means, K-Medoids, Fuzzy C-Means, and GMM all exhibited enhanced clustering performance with increased skewness. For the EL dataset with DR, points were relocated on a nine-dimensional hypersphere with a radius equal to $\frac{1}{30}$ of the cluster size. Increasing skewness resulted in noticeable improvements in CVI metrics across all algorithms. The results are summarized in Figure 17 (without DR) and Figure 18 (with DR). Skewness adjustments revealed irregular behavior in DI, likely due to its sensitivity to cluster shape distortions. For skewed datasets, it is recommended to avoid DI and instead use SH, XB, CH, or DB.

- Subclustering; For the EL dataset without DR, the threshold for all algorithms was set to $\frac{1}{3}$ of the cluster’s radius, except for GMM, which used $\frac{1}{4}$. K-Medoids and GMM showed unexpected improvements across all CVIs, while K-Means exhibited stable Dunn’s Index initially but a later decline, and Fuzzy C-Means displayed inconsistent CVI behavior. For the EL dataset with DR, thresholds for all algorithms were set to $\frac{1}{4}$ of the cluster’s radius. K-Means and GMM demonstrated improvements across all CVIs, while for K-Medoids and Fuzzy C-Means, Dunn’s Index deteriorated instead of improving. The results are summarized in Figure 19 (without DR) and Figure 20 (with DR).
  Subclustering affected only DI, which deteriorated as the minimum inter-cluster distance decreased due to the formation of subclusters. This makes DI suitable for datasets with subclustering.

For outlier removal, prior study [10] recommended DI, while this study highlights CH and DB as more suitable metrics. Similarly, for differential density, prior research endorsed all CVIs except DI, whereas this study finds both DI and DB to be stable and less effective under density changes. However, the results align with previous work regarding the behavior of CVIs for overlapping profiles, kurtosis, skewness, and subclustering.

## 4. Interpretability Analysis

Clustering algorithms can successfully identify consumption patterns and segment daily load profiles into distinct groups; however, these algorithms do not explain why each data point was assigned to a particular cluster [34,35,36,37,38]. This lack of transparency obscures the criteria used to define the boundaries between clusters, making it unclear why certain data points are grouped together. Consequently, stakeholders and domain experts have difficulty verifying the relevance of discovered patterns, refining the clustering process, or addressing anomalies within individual load profiles. Ultimately, without interpretability, clustering results remain opaque “black box” outcomes, limiting their practical utility for making informed decisions about energy management, consumer behavior analysis, or targeted interventions in load demand optimization. To overcome this limitation, two decision-tree-based models were employed: an axis-aligned decision tree and a sparse oblique decision tree. These models offer human-readable rules and graphical representations that emphasize the defining characteristics of each cluster.

Axis-aligned decision tree forms decision boundaries by splitting on individual 15 min intervals within the electric load data. Each node in the tree selects a specific time interval (e.g., 06:30–06:45) and checks whether consumption at that interval is above or below a threshold (i.e., IF/ELSE splits). This structure yields straightforward, interpretable conditions highlighting key consumption periods—for instance, early morning vs. peak evening usage. In contrast, the sparse oblique decision tree is particularly suited to high-dimensional data, because it constructs decision boundaries using linear combinations of multiple intervals. To ensure interpretability, L1 regularization is applied to penalize large coefficients, zeroing out less-relevant time intervals, while a sparsity constraint limits the number of intervals at each split. This keeps the model’s complexity manageable, while capturing the most influential consumption patterns driving cluster distinctions.

Despite their differences, both the axis-aligned and sparse oblique trees produce “IF-THEN” rules to explain cluster assignments. The sparse oblique model expresses these rules as:

$$\sum_{i}{\mathrm{Coefficient}}_{i}\times \mathrm{Consumption}\;\mathrm{at}\;{\mathrm{Time}}_{i}+\mathrm{Intercept}\leq \mathrm{Threshold}$$

where positive coefficients indicate that higher consumption makes the condition harder to satisfy, and negative coefficients do the opposite. By contrast, the axis-aligned tree uses simple threshold-based comparisons on individual intervals:

ConsumptionatTimei≤Threshold

or

ConsumptionatTimei>Threshold

From these rules, several key behavioral patterns emerge, such as morning-active households (high usage from 06:30 to 10:30), evening-active households (18:00 to 22:00), overnight appliance use (00:00 to 05:00), and midday activity (around 11:30 and 14:00). Because PCA was used for dimensionality reduction, a feature-mapping step ensured that each principal component could be traced back to its original 15 min intervals.

**Figure 21:**
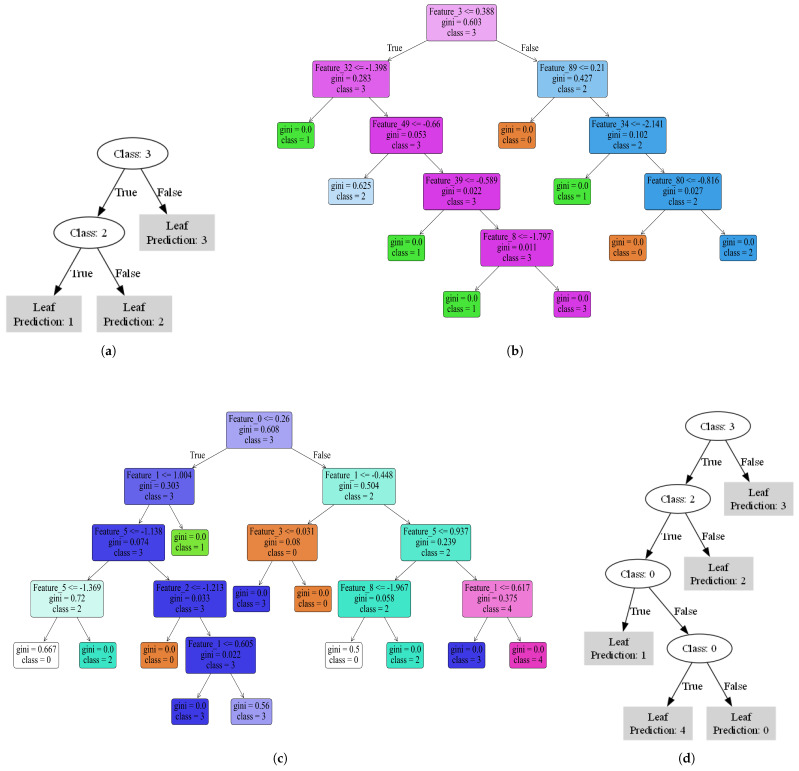
Axis-aligned tree and sparse oblique decision tree results using GMM on the EL dataset without and with dimensionality reduction: (**a**) sparse oblique on EL without DR, (**b**) axis-aligned on EL without DR, (**c**) axis-aligned on EL with DR, and (**d**) sparse oblique on EL with DR.

To provide additional clarity on these interpretability results for residential electricity usage profiles, Figure 21 evaluates the interpretability of clustering results obtained using the axis-aligned decision tree and the sparse oblique decision tree for GMM clustering on the EL dataset. The analysis focuses on the number of rules generated by each model, their ability to explain clusters, and their role in defining the meaning of each cluster, first without DR and then with DR.

Without DR, the axis-aligned decision tree Figure 21b generated nine distinct rules to explain all four clusters, providing detailed coverage. For instance, one rule classified Cluster 0 by relying on splits for Feature_3 ≤ 0.388 (corresponding to consumption at 00:45) and Feature_89 ≤ 0.21 (corresponding to consumption at 22:15). This interpretation indicates that Cluster 0 represents consumers with notable electricity usage late at night (00:45) but minimal consumption late in the evening (22:15). Following this way of interpreting, the rule is generalizable to interpret all the trees, allowing for meaningful insights into the specific behaviors that define each cluster. These single-feature thresholds ensured interpretability and allowed clusters to be defined in terms of residential electricity demand profiles, such as distinguishing between consumers with early morning vs. late-night energy usage patterns. In contrast, the sparse oblique decision tree in Figure 21a explained three of the four clusters using only three rules. While more compact, these oblique splits were less interpretable and failed to explain one cluster. Nevertheless, the rules provided meaningful insights into combined energy usage patterns, highlighting the trade-off between simplicity and coverage. When DR was applied, the axis-aligned tree produced 12 rules to explain all 5 clusters (see Figure 21c). Despite the increased rule count, these rules remained clear, mapping clusters to specific behaviors like weekday midday usage or consistent energy consumption throughout the day. Meanwhile, the sparse oblique tree explained all five clusters with just five rules (see Figure 21d). These compact rules relied on broader patterns, effectively distinguishing high-consumption users from those with minimal fluctuations but sacrificing granularity.

From Table 2, it is evident that in the case of K-Means clustering, cluster 4 consistently appeared as an outlier both without DR and with DR. However, the trees differed in their ability to explain this outlier. Without DR, the sparse oblique decision tree failed to explain cluster 4, leaving it unrepresented. Conversely, the axis-aligned decision tree successfully explained this outlier. With DR applied, the results were reversed: the sparse oblique tree succeeded in explaining cluster 4, while the axis-aligned tree failed to do so. This inconsistency in handling outliers indicates that neither tree is entirely reliable in capturing and explaining outliers when they exist in the dataset. These findings highlight a critical limitation of both models and underscore the need for more robust methods to address outliers in clustering explanations. Table 2 also shows that the axis-aligned tree often explained all clusters or left only one unexplained, demonstrating its robustness in capturing consumption patterns across different clustering algorithms. While it is true that the axis-aligned tree achieves better F1 scores overall, as highlighted in Table 3, this comes at the cost of generating more complex rule sets and larger decision tree graphs, as shown in Appendix A. Conversely, the sparse oblique tree balances its slightly lower F1 performance by providing fewer and simpler rules, which are more concise and interpretable.

The axis-aligned tree’s reliance on threshold-based splits for individual time intervals often results in an exponential increase in the number of rules as the number of clusters grows. For instance, the sparse oblique tree generated up to 9 rules for K-Means, compared to 18 rules by the axis-aligned tree on the EL dataset without DR. This complexity made the axis-aligned tree harder to interpret in larger datasets, whereas the sparse oblique tree maintained simplicity by leveraging linear combinations of features. Conversely, the sparse oblique tree, with careful tuning of parameters like sparsity and lambda, provides a viable alternative, offering concise, interpretable rules at the cost of occasionally missing clusters. However, as evidenced by its failure to explain cluster 4 without DR, the sparse oblique tree also suffers from reliability issues when outliers are present. Future research should investigate optimizing these models to balance interpretability and cluster coverage, especially in complex datasets with outliers.

To make the interpretation more tangible, a single representative example is selected based on rule simplicity to illustrate how decision tree rules explain clustering assignments. Given that 16 different decision trees (8 axis-aligned and 8 sparse oblique) were generated across multiple clustering models and dataset configurations, analyzing each one in detail would be impractical. Below, Table 4 presents an excerpt from the sparse oblique decision tree applied to GMM clustering on the EL dataset without dimensionality reduction, with expanded interpretations that link threshold splits to real-world household behaviors.

This example illustrates how decision tree rules translate clustering assignments into real-world electricity usage behaviors, providing a more transparent and actionable interpretation of consumer load profiles. By revealing that small changes in specific time slots can move a household from one cluster to another, the rules highlight nuanced differences in daily routines, such as how early residents start using appliances or whether certain devices run overnight. In the context of real-world applicability, these interpretable rules can help energy managers, utility providers, and policymakers design targeted initiatives. For instance, households showing steady overnight consumption could benefit from demand response programs that encourage shifting appliance use to lower-cost periods, or they might be prime candidates for time-of-use tariffs that encourage energy usage alignment with off-peak hours. Clusters with higher morning usage might receive energy-efficiency advisories promoting more efficient breakfast appliances or thermostats that manage early heating loads. Moreover, dynamic pricing strategies can be tailored to each cluster’s characteristic load pattern, ensuring more effective peak load reduction and improved consumer cost savings.

## 5. Conclusions

This study evaluated the effectiveness of various clustering algorithms in segmenting residential electricity consumption profiles while addressing their interpretability using decision-tree-based models. By comparing hard clustering methods (K-Means, K-Medoids) with soft clustering techniques (Fuzzy C-Means, Gaussian Mixture Models), the research provided insights into how clustering performance varies based on intra-cluster and inter-cluster characteristics. The results demonstrated that soft clustering methods, particularly GMM, were more effective at capturing overlapping clusters and subtle variations in consumption behaviors, while hard clustering algorithms struggled with boundary definition and outlier sensitivity. These findings become increasingly relevant given the widespread adoption of sensor-based monitoring systems, such as smart meters and IoT devices, which significantly enhance the quality and granularity of electricity consumption data. By providing more detailed and accurate load profiles, these sensor technologies allow clustering algorithms to better identify subtle variations in consumption behaviors, further supporting effective energy segmentation and targeted management strategies. Beyond clustering performance, this study analyzed the impact of data characteristics on clustering validation indices (CVIs), revealing important patterns. Outlier removal had minimal impact on SH, DI, and XB but improved CH, while DB deteriorated unexpectedly, highlighting its sensitivity to inter-cluster separation. Overlapping clusters negatively affected XB in K-Medoids but improved all other CVIs, reinforcing the reliability of SH, CH, and DI in such scenarios. Increased cluster density had a positive impact on SH, CH, and XB, while DI and DB remained largely unaffected, indicating their limited responsiveness to density-related variations. Regarding intra-cluster characteristics, higher central kurtosis improved all CVIs, as clustering became more compact. However, skewness adjustments revealed irregular behavior in DI, suggesting it is less reliable for skewed datasets, whereas SH, XB, CH, and DB remained robust. Subclustering significantly deteriorated DI, confirming its sensitivity to minimum inter-cluster distances, while other CVIs performed more consistently. A key aspect of this study was assessing the effect of dimensionality reduction (DR) on clustering performance. Results showed that dimensionality reduction had minimal impact on clustering quality, as the relative ranking of clustering algorithms and the behavior of CVIs remained stable. However, in some cases, DR improved cluster separability, particularly for overlapping profiles and differential density settings, reinforcing its utility in high-dimensional datasets. This indicates that while dimensionality reduction can help in improving computational efficiency, it does not significantly alter the fundamental clustering structure, suggesting that the choice to apply DR should be based on the trade-off between interpretability and information retention. To enhance interpretability, this study introduced axis-aligned and sparse oblique decision trees to explain cluster assignments. The analysis showed that while axis-aligned decision trees provided complete cluster coverage, their rule complexity increased significantly with the number of clusters. In contrast, sparse oblique trees produced simpler rules but occasionally failed to classify certain clusters. This trade-off between interpretability and completeness underscores the importance of selecting an appropriate explainability framework based on the level of granularity required for decision making. Beyond methodological findings, this study has practical implications for sensor-driven energy management. The interpretable decision rules generated from clustering analyses of sensor-collected data provide a foundation for utility providers and policymakers to develop targeted demand-side strategies. For example, clustering sensor data can reveal groups of households with consistent overnight electricity usage, making them ideal candidates for time-of-use pricing or demand response incentives. Similarly, identifying clusters of households with high electricity usage during morning peak hours allows utility providers to promote energy-efficient appliances or encourage shifting energy-intensive tasks to off-peak periods. Additionally, dynamic pricing models based on clusters identified through analysis of sensor measurements could enhance grid efficiency and reduce consumer energy costs. While these findings contribute to a more explainable approach to clustering in energy data, certain limitations should be acknowledged. The study was conducted on a specific dataset with a fixed set of clustering algorithms and validation metrics, and future research could expand on these results by testing additional clustering techniques or applying the framework to larger and more diverse datasets. Additionally, improving decision tree optimization methods to maintain both interpretability and accuracy remains an open challenge. Ultimately, this research underscores the importance of both clustering accuracy and explainability in energy profiling. By integrating interpretable machine learning techniques with clustering analysis of sensor-collected data, this study provides a framework for transparent, data-driven decision making in electricity demand analysis. Future work should continue refining explainable AI approaches to improve energy forecasting, enhance demand response planning, and support the transition to more intelligent and adaptive energy systems.

## Figures and Tables

**Table 1:** Mapping of principal components to top features and time intervals.

PC (Feature Index)	Top Contributing Features	Time Intervals
PC1	Features {3,4,5,6,7}	00:45, 01:00, 01:15, 01:30, 01:45
PC2	Features {79,80,82,83,84}	19:45, 20:00, 20:30, 20:45, 21:00
PC3	Features {39,40,76,77, 78}	09:45, 10:00, 19:00, 19:15, 19:30
PC4	Features {69,70,71,72, 73}	17:15, 17:30, 17:45, 18:00, 18:15
PC5	Features {27,28,29, 30, 48}	06:45, 07:00, 07:15, 07:30, 12:00
PC6	Features {0,1,69, 70, 72}	00:00, 00:15, 17:15, 17:30, 18:00
PC7	Features {89,90,91, 92,52}	22:15, 22:30, 22:45, 23:00, 13:00
PC8	Features {51,52,50, 49, 88}	12:45, 13:00, 12:30, 12:15, 22:00
PC9	Features {23,24,22, 88, 87}	05:45, 06:00, 05:30, 22:00, 21:45

**Table 2:** Interpretability results for sparse oblique vs. axis-aligned decision trees on the EL dataset with and without dimensionality reduction for different clustering algorithms.

Algorithm	Model	Clusters Explained	Clusters Unexplained
Without Dimensionality Reduction			
K-Means	Sparse Oblique Tree	6/8	3, 4
	Axis-Aligned Tree	All 8	None
K-Medoids	Sparse Oblique Tree	7/9	0,9
	Axis-Aligned Tree	8/9	7
Fuzzy C-Means	Sparse Oblique Tree	2/3	0
	Axis-Aligned Tree	All 3	None
GMM	Sparse Oblique Tree	3/4	0
	Axis-Aligned Tree	All 4	None
With Dimensionality Reduction			
K-Means	Sparse Oblique Tree	All 8	None
	Axis-Aligned Tree	7/8	4
K-Medoids	Sparse Oblique Tree	7/8	0
	Axis-Aligned Tree	All 8	None
Fuzzy C-Means	Sparse Oblique Tree	2/3	1
	Axis-Aligned Tree	All 3	None
GMM	Sparse Oblique Tree	All 5	None
	Axis-Aligned Tree	All 5	None

**Table 3:** F1 score comparison for sparse oblique vs. axis-aligned decision trees on the EL dataset with and without dimensionality reduction for different clustering algorithms.

Algorithm	Sparse Oblique Decision Tree F1 Score	Axis-Aligned Decision Tree F1 Score
Without Dimensionality Reduction		
K-Means	0.692	0.913
K-Medoids	0.766	0.739
Fuzzy C-Means	0.613	0.992
GMM	0.681	0.991
With Dimensionality Reduction		
K-Means	0.907	0.846
K-Medoids	0.655	0.902
Fuzzy C-Means	0.616	1.000
GMM	0.951	0.964

**Table 4:** Interpretation of IF/ELSE rules of axis-aligned decision tree for GMM on the EL dataset without dimensionality reduction, linking thresholds to real-world household behaviors.

Rule	Conditions	Predicted Cluster	Interpretation
1	00:45 ≤ 0.39 08:00 ≤ −1.40	Cluster 1	Very low consumption from midnight to early morning and extremely low in the morning.Real-world implication: Households that barely use electricity overnight, possibly indicating no late-night appliances (e.g., washing machines or dishwashers) and minimal morning routines (e.g., inhabitants leave early).
2	00:45 ≤ 0.39 08:00 > −1.40 12:15 ≤ −0.66	Cluster 2	Slightly higher morning usage, moderate midday usage.Real-world implication: Households with moderate breakfast routines but still low midday activity; could be working families with children who leave by mid-morning.
3	00:45 ≤ 0.39 08:00 > −1.40 12:15 > −0.66 09:45 ≤ −0.59	Cluster 1	Closer thresholds between morning and midday usage lead back to Cluster 1.Real-world implication: Illustrates how small differences in the 09:45 slot can switch a household between two morning-centric clusters, capturing subtle variations in morning routines.
4	09:45 > −0.59 02:00 ≤ −1.80	Cluster 1	Extremely low consumption at 02:00 but higher mid-morning consumption.Real-world implication: Possibly households that shut off most devices before bed but wake up with a substantial breakfast or early appliance use (e.g., coffee machines, toasters).
5	02:00 > −1.80	Cluster 3	Slightly higher overnight usage.Real-world implication: Households that keep some devices running overnight (e.g., fridge plus entertainment devices or charging stations).
6	00:45 > 0.39 22:15 ≤ 0.21	Cluster 0	More consumption after midnight but low late-evening usage.Real-world implication: Households that shift certain appliance usage to shortly after midnight but reduce activities earlier in the evening, indicating evening routines that end relatively early.
7	22:15 > 0.21 08:30 ≤ −2.14	Cluster 1	Late-evening changes trigger very low morning usage.Real-world implication: Possibly households with moderate to high consumption around 22:15, but extremely low consumption by early morning, suggesting that nighttime routines end abruptly, reducing load well before 08:30.
8	08:30 > −2.14 20:00 ≤ −0.82	Cluster 0	Adjusted early-evening consumption leads back to Cluster 0.Real-world implication: Households that may be active enough in the morning to exceed the lower threshold, but remain below the early-evening usage cut-off, indicating little to no significant consumption around 20:00.
9	20:00 > −0.82	Cluster 2	Slightly higher evening consumption sets Cluster 2.Real-world implication: Households that exhibit more pronounced evening activity (e.g., cooking dinner or using entertainment devices) around or after 20:00, pushing them into a higher-consumption cluster.

**Figure A1:**
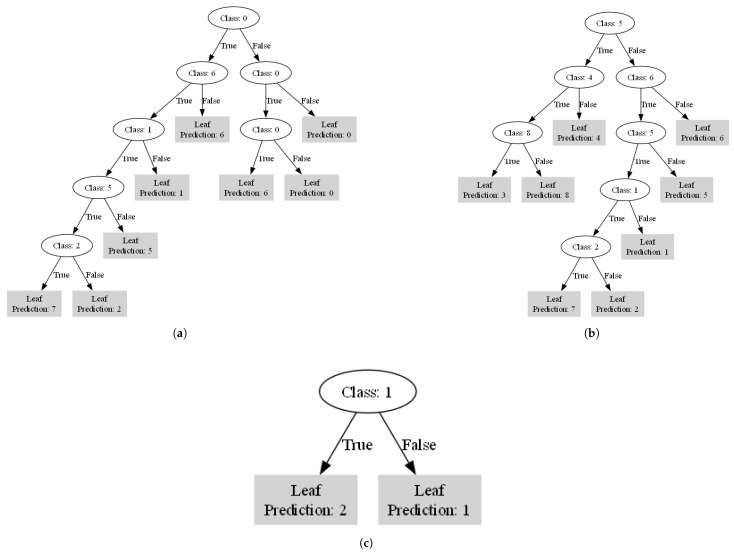
Sparse oblique decision tree using different algorithms on the EL dataset without dimensionality reduction: (**a**) K-Means, (**b**) K-Medoids, (**c**) Fuzzy C-Means.

**Figure A2:**
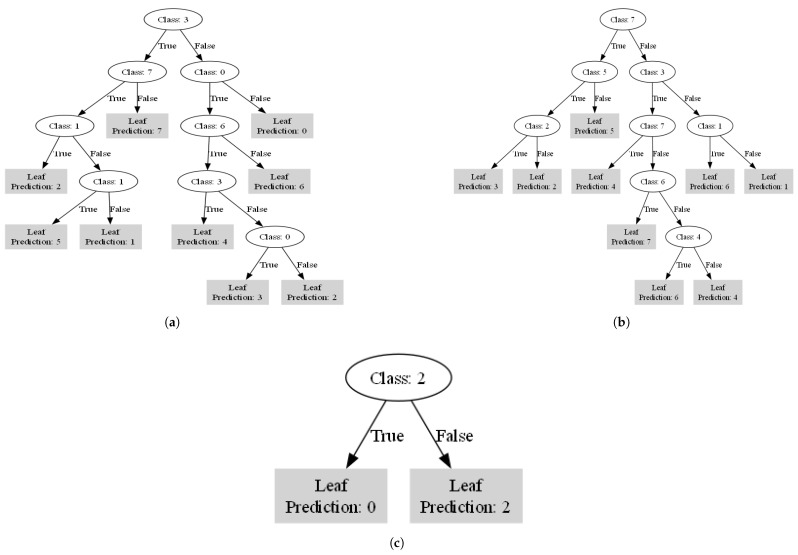
Sparse oblique decision tree using different algorithms on the EL dataset with dimensionality reduction: (**a**) K-Means, (**b**) K-Medoids, (**c**) Fuzzy C-Means.

**Figure A3:**
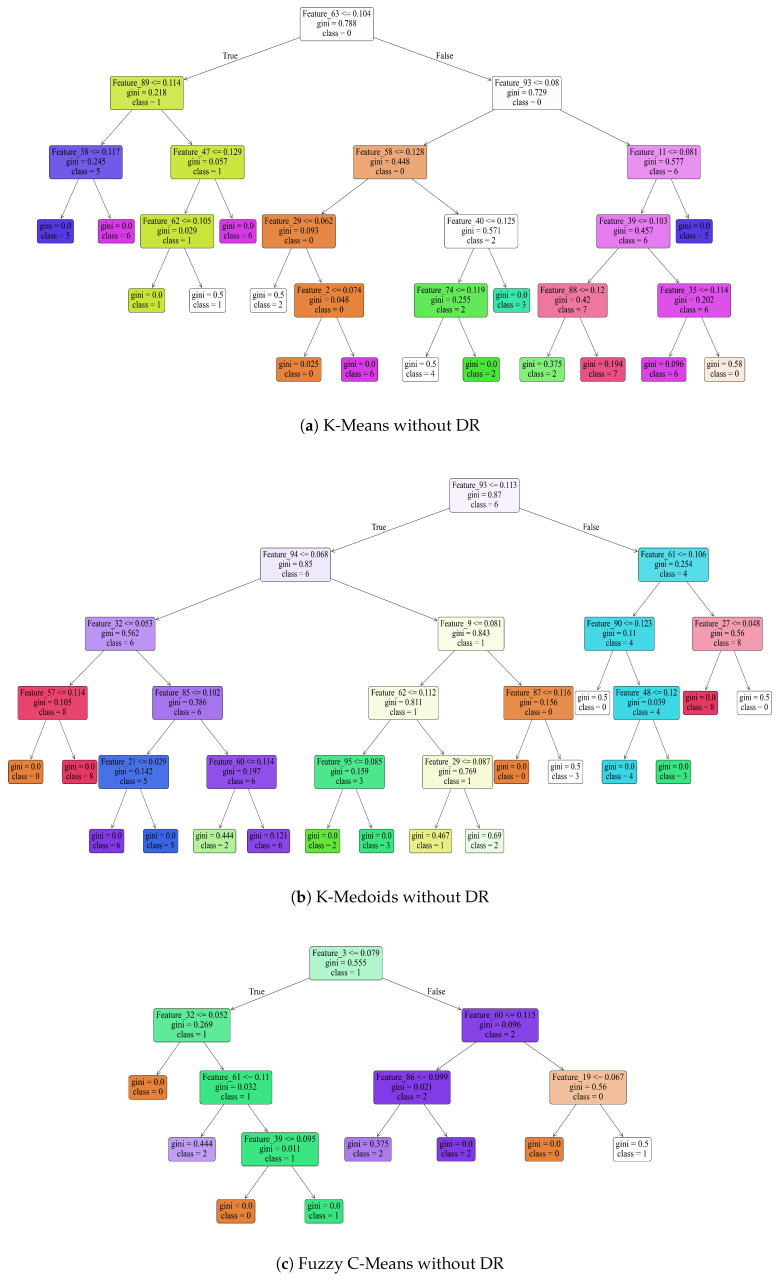
Axis-aligned tree using different algorithms on the EL dataset without dimensionality reduction: (**a**) K-Means, (**b**) K-Medoids, (**c**) Fuzzy C-Means.

**Figure A4:**
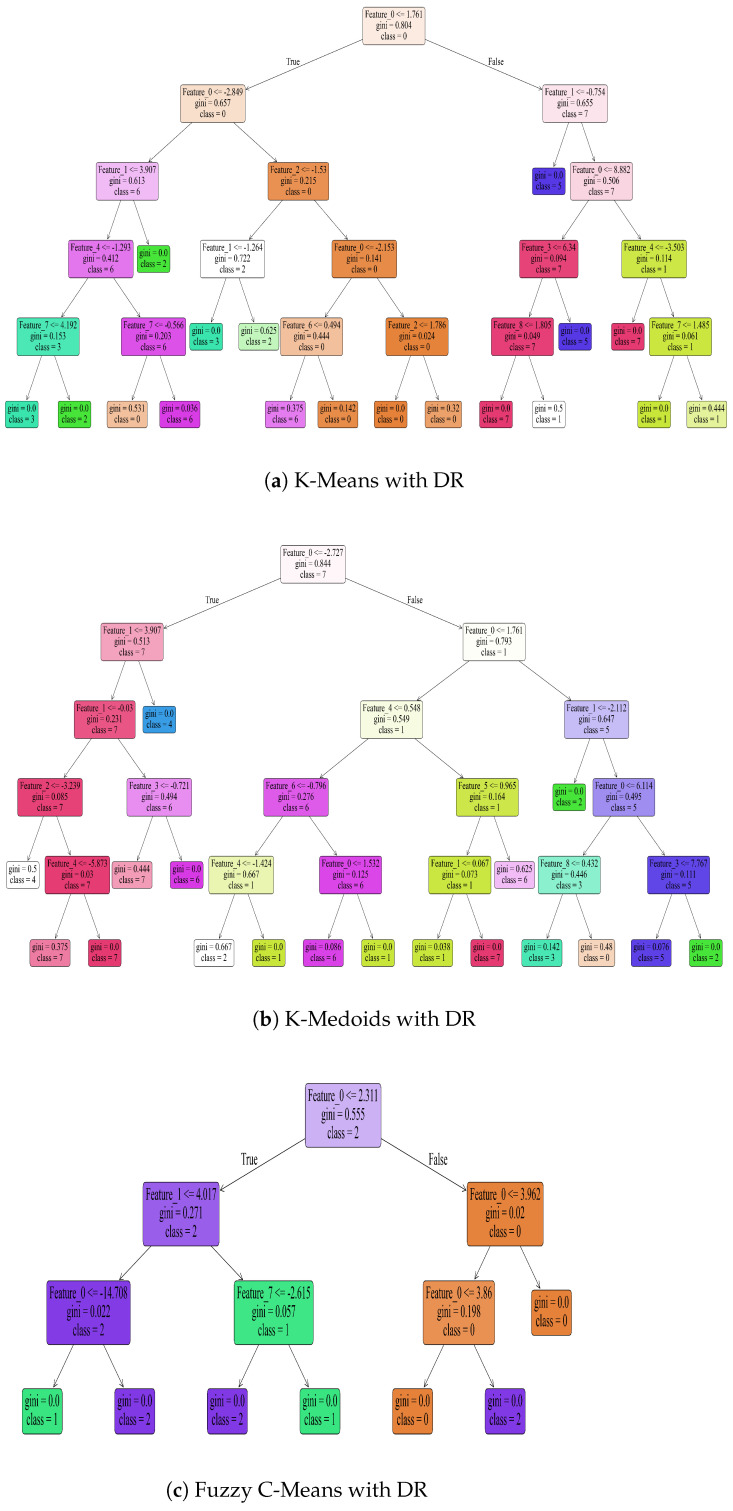
Axis-aligned tree using different algorithms on the EL dataset with dimensionality reduction: (**a**) K-Means, (**b**) K-Medoids, (**c**) Fuzzy C-Means.

## Data Availability

Dataset is contained within the paper.

## Abbreviations

The following abbreviations are used in this study:

DR	Dimensionality reduction
GMM	Gaussian Mixture Model
CVI	Clustering validation index
PCA	Principal Component Analysis
